# Foraging conditions for breeding penguins improve with distance from colony and progression of the breeding season at the South Orkney Islands

**DOI:** 10.1186/s40462-021-00261-x

**Published:** 2021-05-04

**Authors:** Jessica Ann Phillips, Annette L. Fayet, Tim Guilford, Fabrizio Manco, Victoria Warwick-Evans, Phil Trathan

**Affiliations:** 1 Department of Zoology, Oxford University, 11a Mansfield Rd, Oxford, OX1 3SZ UK; 2 Anglia Ruskin University, Cambridge Campus, East Rd, Cambridge, CB1 1PT UK; 3 British Antarctic Survey, High Cross, Madingley Rd, Cambridge, CB3 0ET UK

**Keywords:** Chinstrap penguin, Seabird, Foraging, Habitat selection, Index of patch quality, Prey availability, Ashmole’s halo, GPS, TDR

## Abstract

**Background:**

According to central place foraging theory, animals will only increase the distance of their foraging trips if more distant prey patches offer better foraging opportunities. Thus, theory predicts that breeding seabirds in large colonies could create a zone of food depletion around the colony, known as “Ashmole’s halo”. However, seabirds’ decisions to forage at a particular distance are likely also complicated by their breeding stage. After chicks hatch, parents must return frequently to feed their offspring, so may be less likely to visit distant foraging patches, even if their quality is higher. However, the interaction between prey availability, intra-specific competition, and breeding stage on the foraging decisions of seabirds is not well understood. The aim of this study was to address this question in chinstrap penguins *Pygoscelis antarcticus* breeding at a large colony. In particular, we aimed to investigate how breeding stage affects foraging strategy; whether birds foraging far from the colony visit higher quality patches than available locally; and whether there is evidence for intraspecific competition, indicated by prey depletions near the colony increasing over time, and longer foraging trips.

**Methods:**

We used GPS and temperature-depth recorders to track the foraging movements of 221 chinstrap penguins from 4 sites at the South Orkney Islands during incubation and brood. We identified foraging dives and calculated the index of patch quality based on time allocation during the dive to assess the quality of the foraging patch.

**Results:**

We found that chinstrap penguin foraging distance varied between stages, and that trips became shorter as incubation progressed. Although patch quality was lower near the colony than at more distant foraging patches, patch quality near the colony improved over the breeding season.

**Conclusions:**

These results suggest chinstrap penguin foraging strategies are influenced by both breeding stage and prey distribution, and the low patch quality near the colony may be due to a combination of depletion by intraspecific competition but compensated by natural variation in prey. Reduced trip durations towards the end of the incubation period may be due to an increase in food availability, as seabirds time their reproduction so that the period of maximum energy demand in late chick-rearing coincides with maximum resource availability in the environment. This may also explain why patch quality around the colony improved over the breeding season. Overall, our study sheds light on drivers of foraging decisions in colonial seabirds, an important question in foraging ecology.

**Supplementary Information:**

The online version contains supplementary material available at 10.1186/s40462-021-00261-x.

## Background

Many animals are restricted to repeatedly return to a central place following foraging trips [1]. This includes all animals feeding young in the nest [2–4], animals which use burrows as shelter from predators [5–7], social insects foraging for the colony [8, 9], and incubating birds not fed by their partners [10, 11]. This constraint of repeatedly returning to a central place has implications for the foraging sites animals select, as well the degree of prey depletion [12]. However, the drivers of foraging strategies of central place foragers facing dynamic and heterogeneous environments are not fully understood. In particular, how different factors such as breeding stage and prey availability interact to affect foraging strategy remains unclear. Here, we explore these questions in seabirds, which during breeding act as central place foragers, but also need to commute long distances to forage for patchy and ephemeral prey [13, 14].

Central place foraging theory is a special case of optimal foraging theory [15, 16], in which the forager is tied to a specific location and must return to this location after each foraging bout. Central place foraging theory predicts that animals will only increase the distance of their foraging trips if more distant prey patches offer better foraging opportunities (i.e., better prey quality and/or availability) than local prey patches [17–19] as traveling long distances incurs a cost. The theory predicts that they would return to offspring in a minimal amount of time while acquiring the maximum amount of resources by exploiting the nearest prey patch or exploiting a more-distant high quality prey patch [20, 21]. In seabirds, a consequence of preferential feeding close to the colony during breeding is that in large colonies this could create a zone of food depletion around the colony, known as “Ashmole’s halo” [22], which widens as the season progresses [23]. When this occurs, theory predicts that traveling further would likely yield higher reward per unit time, as prey availability would be higher outside of the halo. However, due to the logistical challenge of quantifying successful foraging events in the open ocean, this prediction has rarely been tested. Ashmole [22] also postulated that this prey depletion around colonies would be less likely at higher latitudes where seabirds can time their reproduction to coincide with seasonal abundance of prey availability. Despite this prediction, evidence of “Ashmole’s halo” has been found at higher latitudes as well as in the tropics. In the tropics, Oppel et al. [2] found that masked boobies *Sula dactylatra* from a smaller colony went on shorter duration foraging trips, foraged closer to the colony, had lower energy expenditure, and higher nest survival than birds from a larger colony. In the UK, Shoji et al. [24] found that patch quality improved with distance from the colony in razorbills *Alca torda*. In Canada, Birt et al. [25] found that the density of bottom fish increased with distance from a double-crested cormorant *Phalacrocorax auritus* colony, providing direct evidence for prey depletion. In northern Canada, Elliott et al. [26] found that the mass of prey retrieved increased with travel distance from a Brünnich’s guillemot *Uria lomvia* colony, suggesting the birds depleted large prey near the colony. However, the interaction between natural variation in prey availability and intraspecific competition in driving seabird foraging decisions are not well understood. For instance, how seabirds change their foraging behaviour when prey depletion near the colony is compensated or aggravated by natural variation in prey availability has not been investigated previously.

Another potential key driver of foraging strategy is breeding stage. Adults also need to adjust their foraging strategy based on the changing needs of their eggs and chicks [20, 27, 28]. During the incubation phase, one parent must fast as they need to keep their eggs warm and protect them from predation and bad weather while the other feeds, after which they will switch role. The chick-rearing phase is more energetically demanding for parents [29], as they have to feed chicks frequently, both to stop the chicks from starving and because young chicks often cannot ingest large quantities of food in a single feeding [30]. Compared to incubating seabirds, chick-rearing seabirds have been shown to have higher foraging costs [31, 32]; have higher field metabolic rates ([33], e.g. [34]); be more active at night [35]; dive deeper (e.g. [35, 36]); reduce their foraging range (e.g. [37, 38]); and perform foraging trips shorter in distance as well as duration (e.g. [31, 39]). However, how breeding stage interacts with prey availability to affect seabird foraging strategies are not fully understood.

We investigate this interaction using chinstrap penguins breeding at the South Orkney Islands, where krill distributions are highly dynamic [40]. Chinstrap penguins are an excellent model to study how breeding stage and prey availability may interact to drive foraging strategy, because they are central-place foragers and efficient divers [41–43] and breed in a very large population of ~ 960,000 pairs [44], making it easier to detect potential effects of intraspecific competition. Chinstrap penguins mainly consume Antarctic krill [45, 46] and smaller amounts of myctophid fish [47, 48]. After laying in early November [49], parents alternate on long incubation shifts between 5 and 10 days [50] until eggs hatch in late December, when parents take turns brooding the chick and making daily foraging trips. We study penguins between the months of December and February, covering the incubation and brood periods.

We fitted 221 chinstrap penguins with Global Positioning System (GPS) devices and temperature-depth recorders (TDRs) to investigate variation in foraging strategies of breeding chinstrap penguins, focusing in particular on the potential roles of intraspecific competition, prey availability, and breeding stage on foraging distance. More specifically, we wanted to determine 1) how breeding stage affects foraging strategy in chinstrap penguins; 2) whether, as predicted by Ashmole’s theory of intraspecific competition, birds foraging far from the colony visit higher quality patches than available locally; and 3) whether prey depletion near the colony increases over the breeding season, which may cause foraging distances to extend and which would indicate an effect of intraspecific competition on foraging strategies.

## Methods

### Field methods

**Fig. 1:**
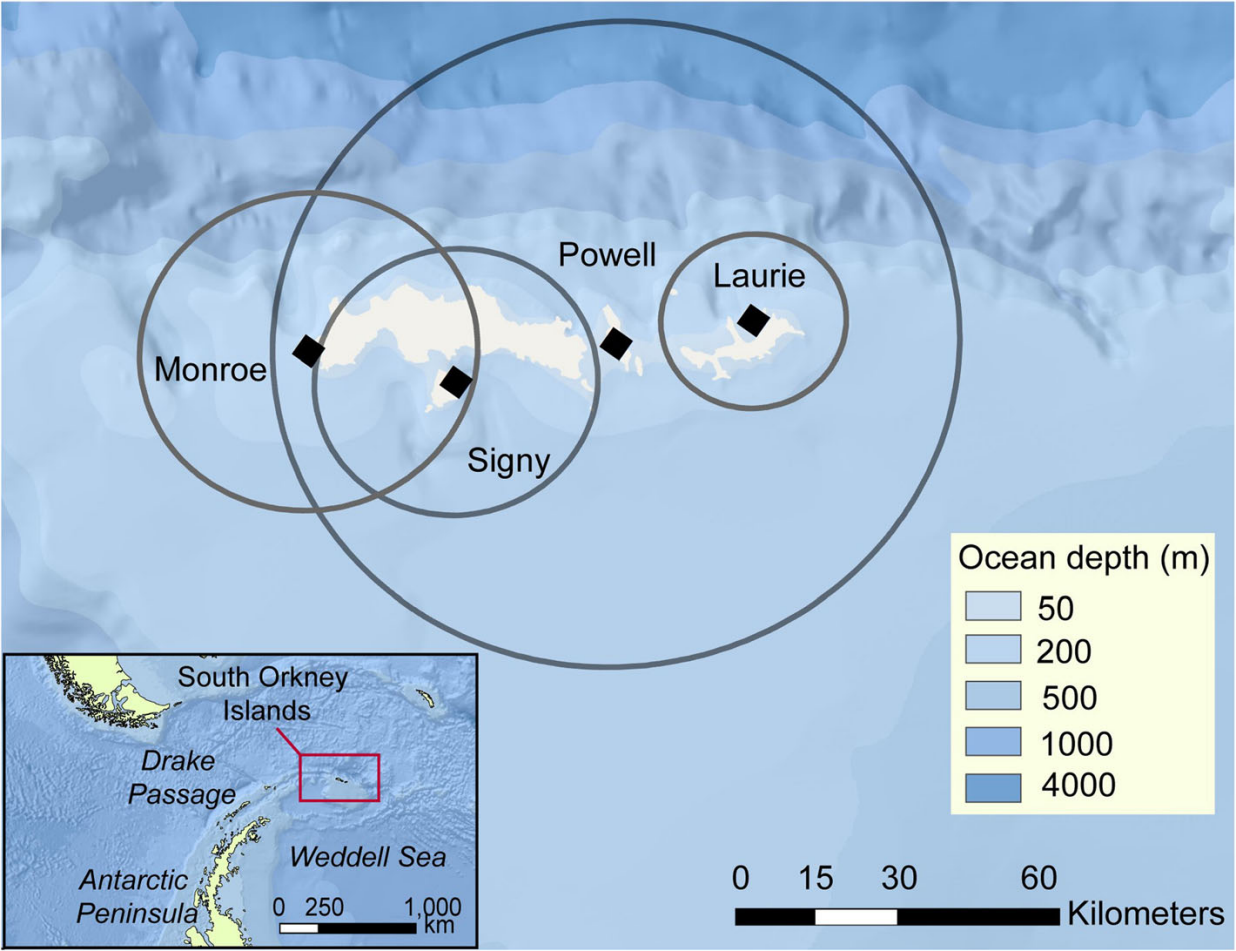
Map of South Orkney Islands. Study colonies are labelled and marked with black diamonds. The circles around each colony have a radius of the median distance of foraging trips up to a day in duration: 17 km at Laurie, 63 km at Powell, 31 km at Monroe, and 26 km at Signy. The base map is from ESRI, Garmin, GEBCO, NOAA and other contributors

All tracking data were collected by scientists and collaborators of the British Antarctic Survey at four of the South Orkney Islands in the Southern Ocean (Fig. 1) between 2011 and 2016: at Cape Geddes (60°41′ S, 44°34′ W) on Laurie island from December 2011 to January 2012, at Powell Island (60°41′ S, 45°02′ W) from December 2013 to January 2014, at Monroe Island (60°36′ S, 46°03′ W) from December 2015 to February 2016, and at Signy Island (60°42′ S, 45°35′ W) from January to February 2016. The population size of breeding chinstrap penguins on the South Orkney Islands is estimated at ~ 960,000 pairs [44]. Signy Island contains~ 19,500 nests [51], Laurie island contains at least 143,800 breeding pairs [52], however the populations of Monroe and Powell Island are unknown [44].

Breeding chinstrap penguins were captured at the nest during the incubation and brood periods, and fitted with a fast acquisition GPS and a TDR device. The TDRs were programmed to record depth every second and the GPS loggers to record location every 4 min – though time intervals were sometimes longer when the GPS could not connect to satellites (e.g., when birds were underwater). The TDRs used were either Lotek LAT 1810 (6 g in air, 38 × 11 mm) or CEFAS G5s (either 2.7 g in air, 31 × 8 mm; or 6.5 g in air, 35 × 12 mm). The GPS loggers used were either Sirtrack™ Fastloc™ 33 F2G133A (38.4 g in air, 65 × 28 × 22 mm with 40 mm whip antenna) or Sirtrack™ Fastloc™ 33 F3G133A (31.0 g in air, 63 × 24 × 22 mm with 40 mm whip antenna). The TDR and GPS loggers were attached with Tesa© tape and 2-part quick-setting glue, as described by Warwick-Evans et al. [53]. Whether chicks were in brood or crèche were recorded at device deployment at all sites and at retrieval in all sites other than Signy. Handling time did not exceed 10 min for deployment and 5 min for retrieval. Tagged penguins included 60 at Laurie Island, 65 at Powell Island, 73 at Monroe Island, and 23 at Signy Island. Devices were retrieved on average after 4.7 ± 0.2 days and the data were downloaded. Potential impacts of logger deployments were not measured directly, but the combined mass of device deployments was always < 1% of the bird’s body mass. In a study investigating tagging impacts on little penguins, the authors found no effect of GPS or TDR attachment on adult body weight change or survival, hatch, fledging or chick growth [54]. Another study on Adélie penguins found no impact of TDR and radio-transmitter attachment on foraging trip duration or nesting success [55]*.*

### Analytical methods

All analyses were undertaken in R 3.5.3 [56] using the ‘DiveMove’ [57], ‘vegan’ [58] and ‘signal’ [59] packages.

First, the GPS output from each penguin was mapped onto the coastline of the South Orkney Islands, obtained from the Scientific Committee for Antarctic Research [60], to identify when the penguin was at sea; all data points while on land were excluded. We defined a foraging trip as a sequence of 10 or more consecutive GPS locations at sea that covered a time span of one hour or more. Trip duration was defined as the interval between the last GPS point on land before the trip and the first GPS point back on land after the trip (see Table 1 for sample sizes).

**Table 1:** Summary of the numbers of chinstrap penguins in each breeding stage which we collected GPS and TDR data from, across four sites at the South Orkney Islands, and the numbers and types of dives performed at each site

	Laurie	Powell	Monroe	Signy	ALL SITES
**NUMBER OF BIRDS,** n	58	52	57	23	190
***BREEDING STAGE***	–	–	–	–	–
incubation, n (%^b^)	22 (38%)	6 (12%)	13 (23%)	9 (39%)	50 (26%)
incubation to brood^a^, n (%)	13 (22%)	9 (17%)	1 (2%)	0 (0%)	23 (12%)
brood, n (%)	23 (40%)	37 (71%)	43 (75%)	14 (61%)	117 (62%)
**NUMBER OF FORAGING TRIPS**, n	155	166	57	51	453
**NUMBER OF DIVES**, n	65,170	64,352	36,479	48,244	214,245
***TYPES OF DIVES***	–	–	–	–	–
foraging, n (%^c^)	14,790 (23%)	15,054 (23%)	6293 (17%)	10,550 (22%)	46,687 (22%)
explore, n (%)	24,515 (38%)	21,695 (34%)	15,842 (43%)	23,634 (49%)	85,686 (40%)
travel, n (%)	25,865 (40%)	27,603 (43%)	14,344 (39%)	14,060 (29%)	81,872 (38%)
**NUMBER OF DIVING BOUTS**, n	2294	2385	980	1180	6839
**NUMBER OF NON-BOUT DIVES**, n	1908	2417	1190	1330	6845

^a^These penguins only had eggs at the nest at deployment and had at least one chick at the nest at retrieval, so the incubation vs brood category could not be assigned

^b^Percentage of birds in this stage at each colony

^c^Percentage of dives which are this type at each colony

#### Identification of Dives

TDR readings from each penguin were truncated to correspond to the start and end times of each foraging trip as determined by the GPS data. To adjust for the drift in TDR pressure readings associated with temperature changes, the depth data were zero-offset corrected [61]. For each separate dive in the foraging trip, we recorded start time, duration, maximum depth, descent time, ascent time, time spent on the surface before the subsequent dive, time spent in the bottom phase of the dive (‘bottom time’), and distance moved up and down during the bottom phase (‘bottom vertical distance’). Bottom vertical distance is a measure of the amount of ‘wiggles’ in the dive that reflects penguins pursuing prey [62–66], and we use it to validate our measure of foraging patch quality (section 4.1). We detected 237,814 dives; 9 dives were excluded because they were far beyond the diving abilities of chinstrap penguins in terms of duration or depth [67] and, thus, presumably the result of TDR malfunction.

In order to determine the number of different dive types performed by chinstrap penguins, we used a machine learning method that employs the Calinski-Harabasz criterion [68] to group dives into relatively distinct clusters. We were particularly interested in identifying foraging dives, so given that prey pursuit [69] and prey capture [42, 66] mainly occurs during the bottom phase of penguins’ dives, we chose bottom time, dive duration, and maximum depth as the criteria for clustering different types of dives [70]. This procedure identified that the optimal number of dive types (i.e., ‘clusters’) was three. We then used k-means clustering to group dives [71] into one of these three groups. We attributed the main cluster of dives with the shallowest depth, shortest duration and shortest bottom time to ‘traveling dives’, dives in which penguins porpoise on the surface to commute to and from foraging areas. We attributed the smallest cluster of dives, with the deepest depth, longest duration, and longest bottom time to ‘foraging dives’, dives in which penguins pursue and manipulate prey at depth. We attributed the medium sized cluster of dives with intermediate depth, intermediate duration and intermediate bottom time as ‘exploratory dives’, dives in which penguins search for prey in the water column but do not successfully capture prey, or capture only small amounts (Table 2). As chinstrap penguins breeding in the South Orkney Islands mainly consume Antarctic krill [46, 48], it is unlikely that this cluster, representing 39% of their dives would be associated with preying on a different type of prey.

**Table 2:** k-mean clustering results (mean (se)) of chinstrap penguins diving data collected with temperature-depth recorders (TDRs) at the South Orkney islands

Dive type	Dive duration (s)	Maximum depth (m)	Bottom time (s)	Number of dives
**Travel**	23.9 (0.04)	5.6 (0.01)	2.3 (0.01)	94,781 (40%)
**Explore**	69.8 (0.04)	22.3 (0.04)	9.3 (0.03)	93,202 (39%)
**Forage**	112.4 (0.08)	61.6 (0.09)	16.0 (0.07)	49,822 (21%)

#### Matching GPS and dive data

For each GPS recording, we calculated the distance to the colony and the time, distance and speed of travel to the next recording. We excluded data from all points where traveling speeds from the previous point and to the following point were both over 10 m/s, as this is not realistic [72] and, thus, indicates GPS device error. We visually inspected the remaining GPS points in each trip to remove other obvious GPS errors, such as single points which were unrealistically distant from the remainder of the track. To further ensure accuracy of the GPS assigned to dives, we also excluded dives that occurred during periods in trips where there was over an hour without GPS data. These three steps resulted in removing 204 of the 98,569 (0.2%) GPS points during foraging trips, and 23,560 of the 237,805 (9.9%) dives during foraging trips. Dives in the remaining time intervals were assigned GPS coordinates by interpolating the GPS data to the start time of each dive, using the Piecewise Cubic Hermite Interpolation ‘pchip’ function in the ‘signal’ [59] package. For each dive, we used the GPS coordinates to determine the distance to the colony.

#### Index of patch quality

Mori et al. [73] developed an ‘index of patch quality’ (IPQ) based on two assumptions: (1) a time-allocation model, i.e. where time spent at depth in a prey patch is a function of both time taken to travel to the prey depth and prey abundance in that patch [12], and (2) the principle of inverse optimality [74], which assumes that foraging dives are optimized to maximize prey intake. IPQ is calculated for each dive as the rate of change in energy gain in relation to the time spent at the bottom phase of the dive [73]:

1 $$ g(t)=a\bullet {t}^x $$

Where *g* is energy gain, *a* is a constant which doesn’t affect the calculation, *t* is the time spent foraging (i.e., in the bottom phase of the dive), and *x* is the IPQ. The time spent on the surface after the dive, *s*, also called surface-pause duration, is a function of dive duration, *u* [75]:

2 $$ s(u)=b{e}^{cu} $$

Where *b* and *c* are constants derived from the relationship between *u* and *s*. Based on Eq. (1, 2) we can derive [75]:

3 $$ x=\frac{\left(1+ bc{e}^{cu}\right)\left(u-\tau \right)}{bc{e}^{cu}+u} $$

Where *τ* is the time taken to travel to the depth of the prey patch. The IPQ has been used as a metric of prey abundance in several marine predators [24, 76–78], and has been shown to correlate with various proxies of prey abundance including with prey mass brought back to the colony in seabirds [75], prey abundance measured by on-board cameras in Weddell seals [77], and krill abundance measured by hydro-acoustic surveys in a study of fur seals [76].

We calculated the IPQ for each foraging dive, except those with a subsequent surface pause greater than 325 s (determined by the inflection point in the frequency of post-dive surface pause graph [63, 79]) because longer pauses were unlikely to indicate the time taken for penguins to recover from the dive. While Elliot et al. [75] estimated the sum of ascent and descent time in the function to calculate IPQ as a linear function of bottom depth, we were able to use the actual ascent and descent time for each dive, which improves accuracy of the estimation of IPQ. We also excluded 7 dives with IPQ greater than 5, as its not realistic for energy gain to increase exponentially with bottom time to the power of five [73, 75]. We calculated the value for the constants b and c using Eq. (2), by running the equation on the dive duration (u) and surface pause duration (s) for all foraging dives, and taking the median values for b and c. We identified ‘diving bouts’ as clusters of foraging dives occurring within less than 325 s of surface time between each other. For each diving bout we recorded the number of dives in the bout, the mean distance to the colony, and mean IPQ. To validate our use of IPQ as an indicator of patch quality, we tested whether as predicted IPQ increased with the number of dives in a bout [73] and with the vertical distance covered at the bottom, which reflects penguins pursuing prey [63] .

#### Statistical analysis

We used linear mixed models (LMM) to validate the IPQ by testing for 1) correlations between dive IPQ and with bottom vertical distance; 2) correlations between bout IPQ (i.e., mean IPQ for all dives in a bout) and the number of dives in the bout, and 3) whether dive IPQ differed between dives which were or were not part of a diving bout. We then used LMMs to test for evidence of an Ashmole’s halo close to the colony by testing if 4) bout IPQ differed with the distance from the colony. We used LMMs to investigate prey depletion near the colony over the whole season by testing if 5) IPQ of bouts near colonies varied with the date of occurrence. For each colony, we took the median of the maximum distance to the colony of all foraging trips that were less than a day in duration, and defined ‘bouts near colony’ as all diving bouts that occurred within this distance. Finally, we used LMMs to test for differences in foraging behaviour between breeding stages by testing if 6) foraging dive depth differed between breeding stages; 7) maximum trip distance differed with breeding stage; and 8) maximum trip distance of foraging trips during the incubation and brood phases differed with the trip start date. We converted all dates into Julian day, and added 365 to dates in January and February so the numbers indicating day would be continuous from December to January. Twenty-three penguins were excluded from the last three models because they had eggs at deployment but had at least one chick at retrieval and, thus, it was not straightforward to assign breeding stage.

For each model, we set penguin identity as a random effect because our dataset contained multiple dives and trips per bird, colony as a fixed effect to account for potential differences across populations, although colony and year are confounded so differences could be attributed to either. To obtain significance values, we used likelihood ratio tests comparing the model of interest with the null model (the same model but without the variable of interest). For models 4 to 8, we used likelihood ratio test to compare the selected model with a model without colony, to determine whether the effect of colony was significant. If the effect of colony was significant, we ran a separate model for each colony. As each penguin only performed one trip at Monroe Island, we did not need to include penguin identity as a random effect for this island, and used a linear model (LM) for this island in model 8. We square-root transformed IPQ in all models to normalize its distribution. All estimates presented in the results section are mean ± standard error (se) unless indicated otherwise.

## Results

Chinstrap penguin foraging trips were predominantly performed in northeast, northwest and southwest directions of the South Orkney Islands. The at-sea distribution of the birds are described in Warwick-Evans et al. [53], here we focus solely on the birds’ foraging behaviour.

### Validation of IPQ

**Fig. 2:**
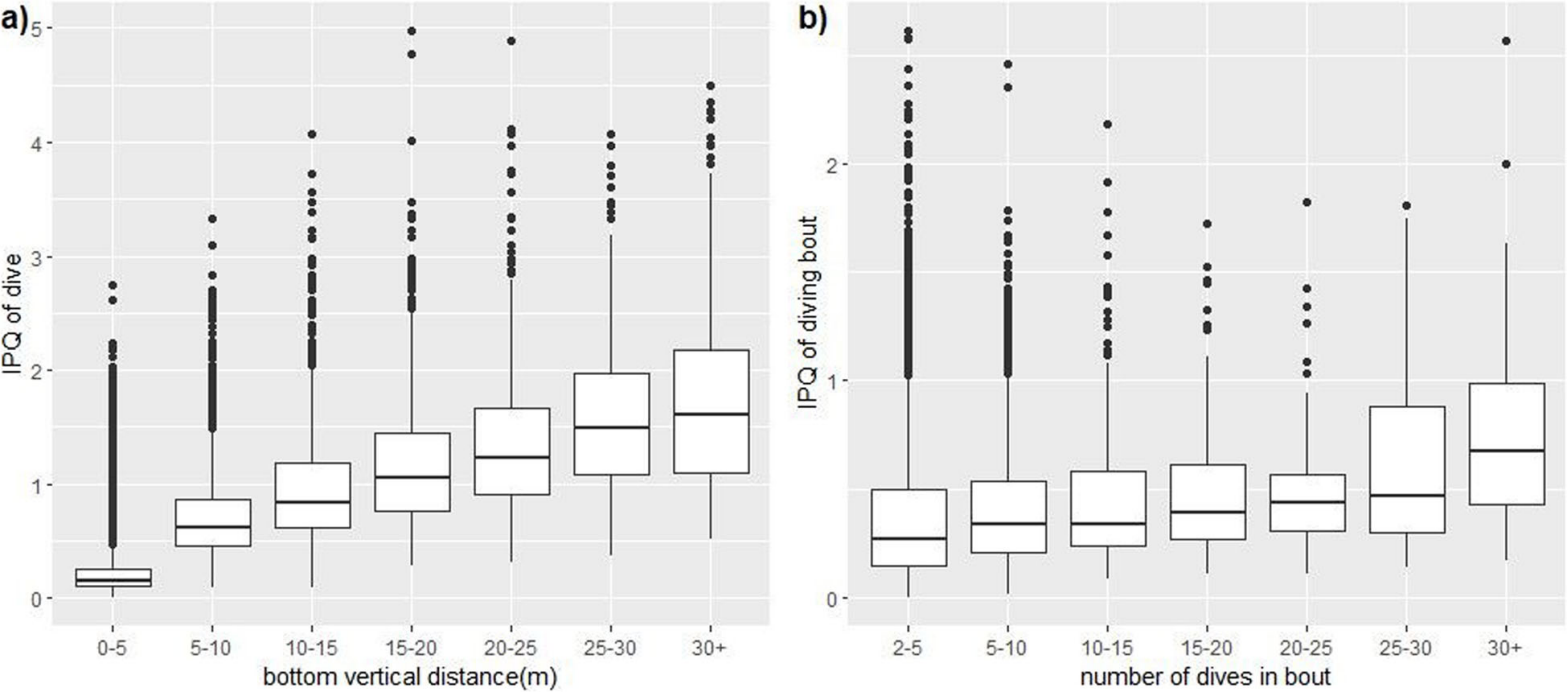
The **a**) bottom vertical distance and IPQ of foraging dives, and **b**) number of dives in foraging bouts versus mean bout IPQ, for chinstrap penguins breeding on the South Orkney Islands

Our validation of the IPQ as a measure of patch quality is three-fold. First, we found that IPQ significantly increased with bottom vertical distance (LMM: slope = 0.0259 ± 0.0001, χ^2^_1_ = 26,876, *p* < 0.0001, Fig. 2a). Second, we found that mean IPQ of a dive bout increased significantly with the number of dives in that bout (LMM: slope = 0.0055 ± 0.0004, χ^2^_1_ = 227.97, *p* < 0.0001, Fig. 2b). Diving bouts lasted on average 16.38 ± 0.42 min and contained an average of 5.83 ± 0.09 dives. Third, we expect diving bouts indicate higher prey abundance, and found that the IPQ of dives in bouts was significantly higher than the IPQ of single dives (in a bout: 0.472 ± 0.003, single: 0.342 ± 0.005, LMM: χ^2^_1_ = 352.97, *p* < 0.0001), validating IPQ.

### Differences between breeding stages

**Fig. 3:**
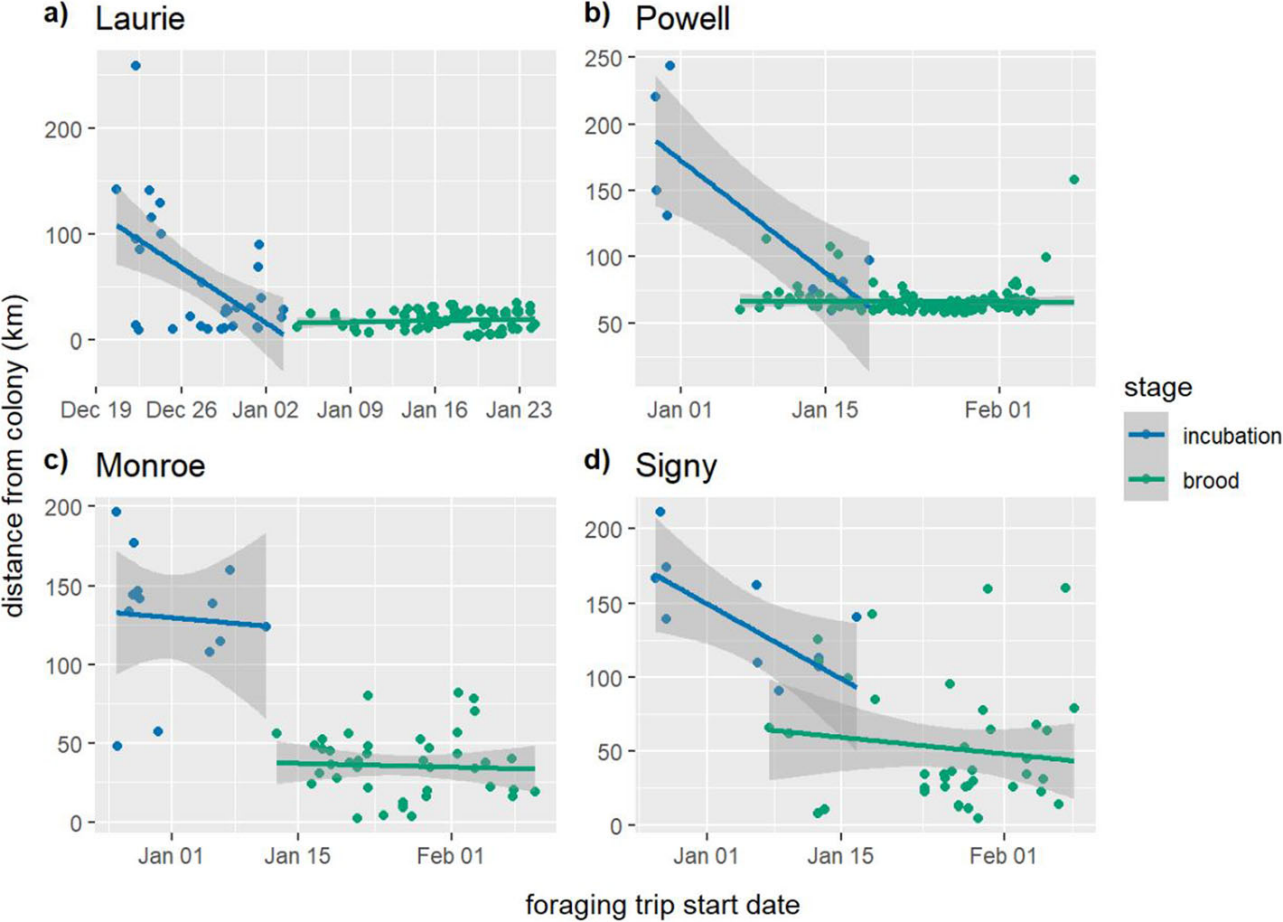
Depth of chinstrap penguin foraging dives during incubation and brood at four colonies in the South Orkney Islands

Overall, foraging dives during incubation were significantly shallower than foraging dives during brood (incubation: 50.67 ± 0.12 m, brood: 68.80 ± 0.13 m, LMM: χ^2^_1_ = 84.44, p < 0.0001), with a significant effect of colony (χ^2^_3_ = 28.24, p < 0.0001). The effect was significant at every colony (Laurie: incubation: 54.38 ± 0.21 m, brood: 70.20 ± 0.22 m, LMM: χ^2^_1_ = 25.58, p < 0.0001; Powell: incubation: 54.26 ± 0.29 m, brood: 73.18 ± 0.22 m, LMM: χ^2^_1_ = 10.84, p < 0.0001; Monroe: incubation: 47.35 ± 0.22, brood: 67.75 ± 0.30 m, LMM: χ^2^_1_ = 38.98, p < 0.0001; Signy: incubation: 45.13 ± 0.15 m, 60.98 ± 0.25 m, LMM: χ^2^_1_ = 13.75, *p* = 0.00021, Fig. 3). Overall, foraging trips during the incubation phase were significantly longer than those during the brood phase (incubation: 94.06 ± 8.21 km, brood: 46.54 ± 1.58 km, LMM: χ^2^_1_ = 105.66, p < 0.0001), with a significant effect of colony (χ^2^_3_ = 70.64, p < 0.0001). The effect was significant at every colony (Laurie: incubation: 54.62 ± 10.58 km, brood: 18.28 ± 0.94 km, LMM: χ^2^_1_ = 12.15, *p* = 0.00049; Powell: incubation: 124.58 ± 22.69 km, brood: 66.45 ± 0.96 km, LMM: χ^2^_1_ = 32.40, *p* < 0.0001; Monroe: incubation: 129.94 ± 11.58 km, brood: 35.73 ± 3.14 km, LM: t = 11.11, p < 0.0001; Signy: incubation: 134.26 ± 12.89 km, brood: 52.01 ± 6.69 km, LMM: χ^2^_1_ = 17.13, *P* < 0.0001).

**Fig. 4:**
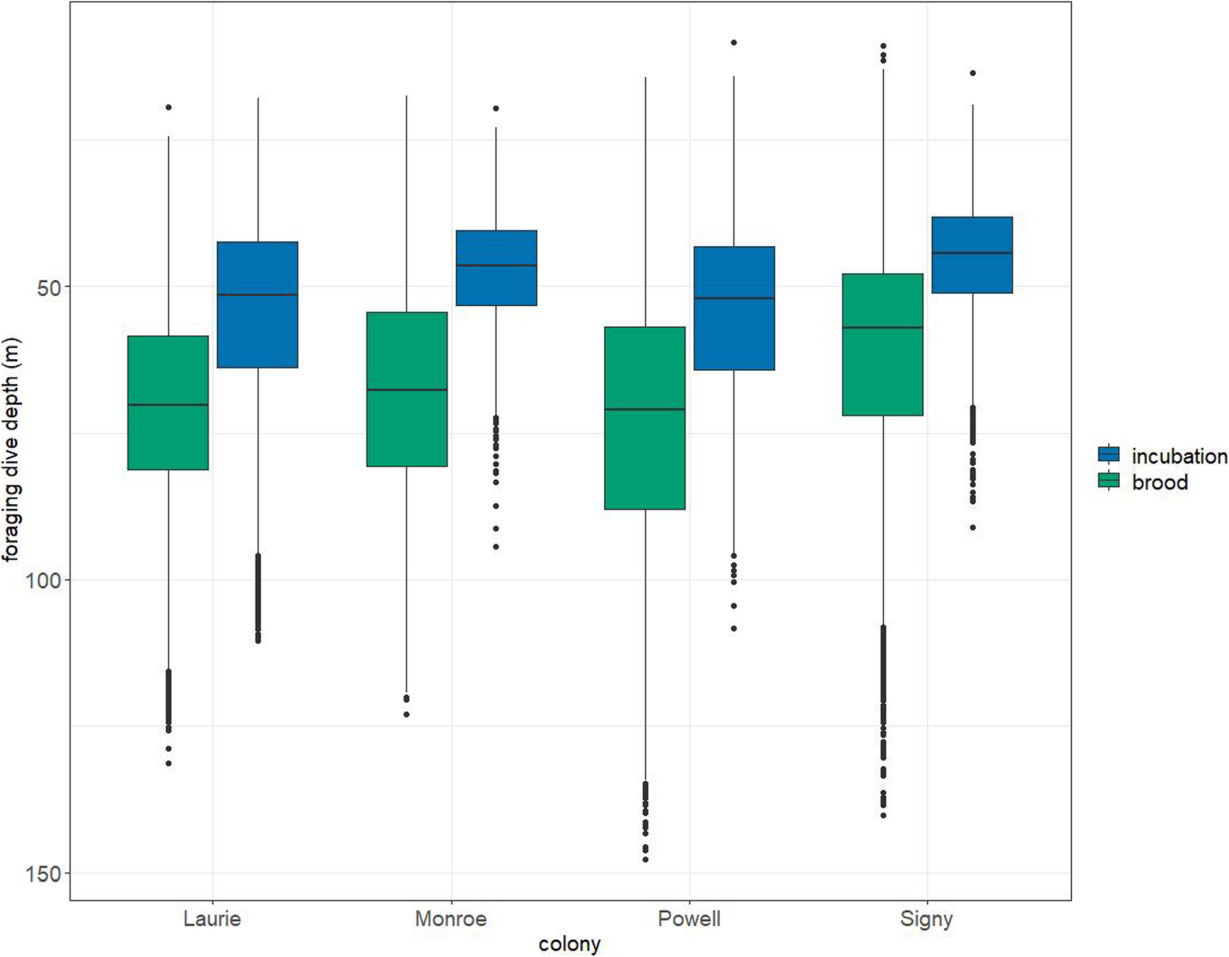
Maximum distance from the colony of foraging trips by start date during incubation and brood at **a**) Laurie Island, **b**) Powell Island, **c**) Monroe Island, and **d**) Signy Island. The shaded areas represent slope standard error

Overall, trip distance during incubation declined significantly with trip start date (LMM: slope = − 0.14 ± 1.30, χ^2^_1_ = 13.89, *p* = 0.00019), but there was a significant effect of colony (χ^2^_3_ = 33.63, P < 0.0001). When the colonies were analysed separately, the relationship was significant for Laurie and Signy (Laurie: LMM: slope = − 7.45 ± 2.22, χ^2^_1_ = 7.82, *p* = 0.0052; Signy: LMM: slope = − 3.67 ± 1.49, χ^2^_1_ = 5.63, *p* = 0.018, Fig. 4) but not the other colonies (Powell: LMM: slope = − 5.2 ± 2.14, χ^2^_1_ = 3.68, *p* = 0.055; Monroe: LM: slope = − 0.72 ± 2.22, t = − 0.32, *p* = 0.75, Fig. 4). Overall, trip distance during brood declined significantly with trip start date (LMM: slope = − 0.039 ± 0.012, χ^2^_1_ = 7.29, *p* = 0.0069), with a significant effect of colony (χ^2^_3_ = 91.20, P < 0.0001). However, when colonies were analysed separately, there was no significant effect at any colony (Laurie: LMM: slope = 0.15 ± 0.24, χ^2^_1_ = 0.43, *p* = 0.51; Powell: LMM: slope = − 0.0094 ± 0.0072, χ^2^_1_ = 1.76, *p* = 0.18; Monroe: LM: slope = − 0.031 ± 0.020, t = − 1.57, *p* = 0.12; Signy: LMM: slope = 0.069 ± 0.047, χ^2^_1_ = 2.12, *p* = 0.15, Fig. 4).

### Prey availability as a determinant of foraging distance

**Fig. 5:**
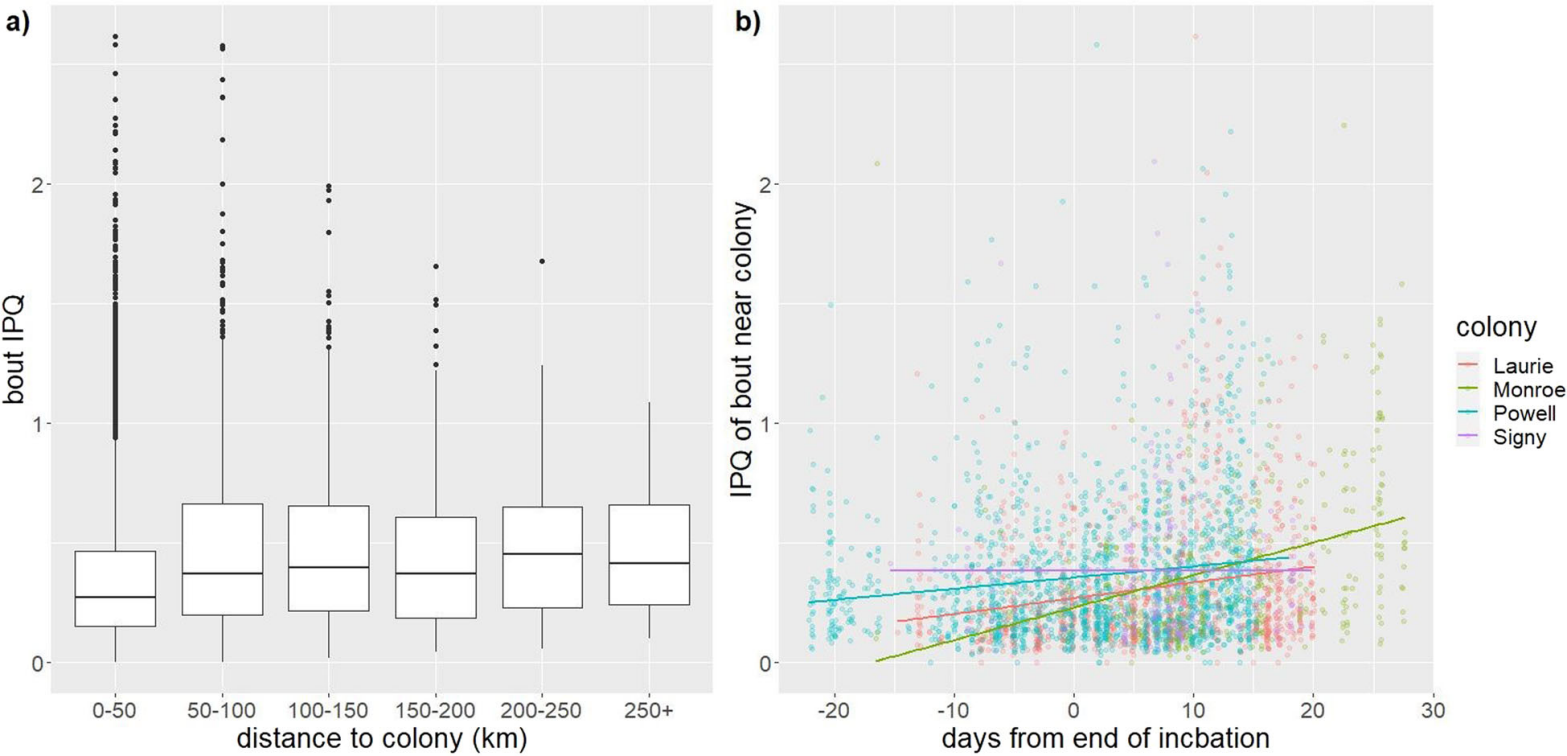
**a**) IPQ of diving bouts against the distance from the colony; **b**) IPQ of bouts near the colony (i.e. within the median distance of trips up to a day in duration: 17 km at Laurie, 63 km at Powell, 31 km at Monroe, and 26 km at Signy) over the breeding season. Where “0” on the x-axis denote the day the last tracked incubation trip ended. Trips that started before the end of the final incubation trip have negative values, and trips that started after that have positive values. The lines represents a linear model fit to the data from each colony

Overall, bout IPQ increased significantly with the distance to colony regardless of breeding stage (LMM: slope = 0.00071 ± 0.000099, χ^2^_1_ = 43.85, *p* < 0.0001, Fig. 5a), with no significant effect of colony (χ^2^_3_ = 7.32, *p* = 0.062) (see Fig. 1 for a kernel density plot of IPQ where penguins foraged). However, this effect was mainly driven by the brood period where IPQ increases significantly with distance to colony (LMM: slope = 0.0015 ± 0.00028, χ^2^_1_ = 28.21, p < 0.0001), with no significant effect of colony (χ^2^_3_ = 0.45, *p* = 0.93). During incubation, while IPQ increased significantly with distance to colony (LMM: slope = 0.00081 ± 0.00012, χ^2^_1_ = 35.82, p < 0.0001), there was a significant effect of colony (χ^2^_3_ = 8.71, *p* = 0.033). When the colonies were analysed separately, the relationship was significant for Laurie (LMM: Laurie: slope = 0.0013 ± 0.00019, χ^2^_1_ = 42.54, p < 0.0001), but not the other colonies (Powell: slope = 0.00032 ± 0.00025, χ^2^_1_ = 1.81, p = 0.18; Monroe: slope = 0.00030 ± 0.00039, χ^2^_1_ = 0.62, *p* = 0.43; Signy: slope = 0.00055 ± 0.00029, χ^2^_1_ = 3.53, *p* = 0.060).

### Prey availability near the colony throughout the breeding season

To investigate whether patch quality near the colony declined over the breeding season, we tested the relationship between the date of occurrence of diving bouts near the colony (i.e. within the median distance of trips up to a day in duration: 17 km at Laurie, 63 km at Powell, 31 km at Monroe, and 26 km at Signy), and their IPQ. We found bout IPQ near the colony increased significantly through the breeding season (LMM: slope = 0.0041 ± 0.00065, χ^2^_1_ = 22.33, p < 0.0001, Fig. 5b) with no significant difference between colonies (χ^2^_3_ = 1.68, *p* = 0.64).

## Discussion

We used a large population tracking dataset to test how breeding stage, prey abundance and intraspecific competition influenced foraging strategies of breeding chinstrap penguins. We found strong evidence that breeding stage influenced foraging strategies in chinstrap penguins. Foraging trips became shorter in distance with the progression of incubation at Laurie and Signy Island. We also found that foraging patch quality increased with increasing distance from the colony at all colonies during brood, and at Laurie during incubation. Finally, we found that patch quality near the colony improved over the breeding season at all colonies.

Breeding stage had a strong impact on foraging tactic. First, penguins travelled further from the colony during incubation than chick rearing at all colonies, in line with numerous other tracking studies of seabirds, including penguins [31, 35, 36, 39, 80, 81]. Thus, when penguins prioritized maximizing energy gain (during incubation) [39], they targeted further foraging grounds, which are likely more profitable as shown by our IPQ measurements. Conversely, when they prioritized minimizing time at sea (during chick-rearing) [39], they targeted closer foraging grounds that had a lower IPQ and were therefore likely less profitable [39]. Differences between breeding stages were not only visible in terms of spatial distribution however, we also found differences in the birds’ diving behaviour. Foraging dives were significantly deeper during chick-rearing compared to foraging dives during incubation at all colonies. Similar findings have been established in Adélie penguins *Pygoscelis adeliae* [82], and king penguins *Aptenodytes patagonicus* [35]. Deeper dives are more energetically costly to birds than shallow dive as they incur higher metabolic costs [83]. This suggests that chinstrap penguins – like other seabirds [35, 36, 84] – reduce foraging effort when their time at sea is less restrictive during incubation, and increase foraging effort when their time at sea is more restrictive during chick-rearing [31]. Foraging at shallower depths may also permit chinstrap penguins more time to search for high quality prey patches during incubation [35, 85, 86]. Alternatively, foraging effort may have to increase during chick rearing due to lower prey availability near the colony. As the survival of penguin chicks is associated with feeding frequency [87, 88], parents must remain close to the nest during chick-rearing to ensure they are fed frequently.

We also observed differences in foraging tactic within a breeding stage. As incubation progressed, birds at Laurie and Signy Island made increasingly shorter trips. This shortening of foraging trips toward the end of incubation leading up to hatching has also been found in albatrosses [81, 89], petrels [90, 91], fulmars [92], Manx shearwaters *Puffinus puffinus* [93], and king penguins [94]. Two hypotheses have been proposed to explain this behaviour [95]: 1) parents predict hatch date based on cues from the egg or from an internal clock, 2) shortening trips are due to seasonal increase in prey availability. The former represents a preparation for a change in parental duties (young chicks need to be fed soon after hatching, therefore parents may want to avoid being on a long trip when the egg hatches), and the latter representing a simple reaction to changing environmental conditions. In a manipulative experiment, Gonzalez-Solis [95] found that petrels use an internal clock to predict hatch date, which they fine tune with signals from the egg. By shortening trips near to hatch, parents thereby ensure chicks are fed soon after hatching, as the incubating bird may be unable to feed the chick after days of fasting whilst sitting [95]. However, we found that the patch quality near the colony increased over the breeding season at all islands, which suggests that the reduction in trip length near hatching may be due to a seasonal upsurge in prey availability. This hypothesis is not mutually exclusive to the scheduling one, and so we cannot rule out that birds also shortened their trips in time for chick hatching [95].

Ashmole [22] suggested that food availability may ultimately limit seabird population size, as the intraspecific competition at colonies with high densities of birds would deplete prey around the colony, and reduce provisioning rates to chicks, which would then impact reproductive success, recruitment rates, and ultimately, colony size. We did find some evidence for a “halo” of lower prey availability near the colony, in the form of lower IPQ closer to the colony at all colonies. However, we did not find any evidence of prey depletion increasing around the South Orkney Islands as the breeding season progressed, in fact we found the patch quality increased over time. Ashmole [22] postulated that seasonal abundance of prey availability in higher-latitudes would make prey depletion around higher-latitude seabird colonies less likely. However evidence of “Ashmole’s halo” has been found in a number of higher-latitude seabird colonies, including razorbills at Skomer Island, UK [24], double-crested cormorant at Prince Edward Island, Canada [25], and Brünnich’s guillemot in Nunavut, northern Canada [26]. This study adds to the evidence of “Ashmole’s halo” existing alongside seasonal upsurge in prey availability. Kokubun et al. [96] found that during brood chinstrap penguins at Barton Peninsula on King George Island foraged further from the colony with time, and attributed this to prey depletion. King George Island and the neighbouring Nelson Island have a breeding chinstrap penguin population estimated at 625,800 pairs [97], smaller than our population of ~ 960,000 pairs [44]. It is therefore unlikely this difference between our results is due to population size. One possible explanation for this difference is that the South Orkney Islands may be in an area with particularly abundant prey, and a similar number of chinstrap penguins would be unable to deplete the prey abundance in this area, whereas they would around King George Island. This is supported by findings based on five summers of krill surveys that the shelf break northwest of the South Orkney Islands is a hotspot for krill concentration and retention [40]. Another explanation could be that the highly dynamic krill distribution near the South Orkney Islands [40] results in the movement and replenishment of Antarctic krill, through flux with the ocean currents and reproduction [98]. This may compensate for prey depletion by intraspecific competition near the colony during breeding, an argument supported by our finding that patch quality increases near the colony as the season progressed.

Seabirds are thought to time their reproduction so that the time of maximum energy demand in late chick-rearing coincides with the period of maximum resource availability in the environment [13]. This is probably why we found that IPQ near the colony increased later in the breeding season. This may also be why adults at Laurie and Signy made shorter trips later on in Incubation, as their energy demands could be met more quickly [95]. Changing foraging ranges throughout breeding may also reduce competition with Adélie penguins where the two species breed sympatrically, such as on Signy Island [99] and Laurie Island [52]. There are ~ 19,500 chinstrap penguin nests and ~ 18,300 Adélie penguin nests on Signy Island [51], and at least ~ 143,800 pairs of chinstrap penguins and ~ 81,000 Adélie penguin nests with eggs on Laurie Island [52]. Clewlow et al. [99] found an allochory of 28 days between chinstrap and Adélie penguins breeding sympatrically on Signy Island caused them to leapfrog each other’s foraging locations and reduced the overlap of their habitat use by 54% over the breeding season. The shortening of foraging trips towards the end of incubation at Signy and Laurie Island may help to reduce overlap in habitat use with sympatric Adélie penguins. While the population sizes of chinstrap penguins at Monroe and Powell Island are unknown [44], penguins travel on average twice as far during trips less than a day in duration at Powell Island compared to any other island, indicating more competition for resources, and/or lower prey availability near Powell Island or during the 2013–2014 season. Trip distances over brood had a lower standard error at Laurie and Powell than Monroe and Signy. This may be partly caused by the smaller sample size of brooding penguin at Signy compared to the other colonies. This could also be associated with the colonies’ proximity to the shelf edge. At Laurie and Powell, located on the east of the South Orkney Islands, most birds used the shelf edge north of the colony. In contrast, birds from Monroe and Powell had further distances to travel to reach the shelf edge to the west of the South Orkney Plateau, which could have led to a greater variation in foraging distance as not all birds travelled to the edge.

However, there may also be benefits to foraging in proximity to other penguins. Sutton et al. [100] found that African penguins *Spheniscus demersus* often forage in proximity to a variety of other predators, and foraging in proximity to other seabirds improved individual foraging success. However, while *Eudyptula minor* little penguins often associated with conspecifics while hunting schooling prey, their foraging gains were similar or smaller compared to solitary foraging on schooling prey, indicating penguins may have to trade off reduced energetic gains from prey against increased likelihood of locating prey items [101]. Furthermore, Sutton et al. [102] found that macaroni penguins did not associate with conspecifics during foraging, and attributed this to the low maneuverability and high prevalence of krill. It is not known whether chinstrap penguins associate with other conspecifics or sympatric penguin species during foraging attempts. Future research shedding light on this question could provide information on whether chinstrap penguins can also benefit from foraging in areas with high concentrations of penguins. We found that bout IPQ increased with the distance from the colony, providing evidence for Ashmole’s halo, and suggesting that chinstrap penguins’ foraging strategies are partly influenced by prey distributions. Our findings support previous studies by Shoji et al. [24], who found that IPQ increased with the distance from the colony in razorbills*.* This result also aligns with other studies which found evidence of Ashmole’s halo, including Birt et al. [25], who found that the density of bottom fish increased with distance from a double-crested cormorant colony, and Elliot et al. [26], who found that the mass of prey items brought back increased with foraging distance in chick-rearing Brünnich’s guillemots. One limitation of our study is that we did not consider potential sex differences. However, previous studies found no intersex differences in chinstrap penguin maximum trip distances [103, 104], time spent on the shelf [104], or the proportion of fish in the diet [104, 105]. Another limitation of our study is that we did not directly measure patch quality, but instead used IPQ as a proxy. However, several studies have found correlations between IPQ and measures of prey abundance [76, 77]. Furthermore, we found correlations between multiple variables likely linked with prey abundance and IPQ. These studies and our own validation suggest that IPQ is an appropriate proxy of patch quality [106]. Another finding which highlights how chinstrap penguins respond to local habitat quality is that the number of dives in a bout was positively correlated with the mean IPQ of the bout, showing that birds spent longer in higher quality patches. This supports optimal diving models, which predict that divers should perform more dives in high quality patches, though it has seldom been tested due to the challenges of measuring patch quality [73]. Likewise, Mori et al. [73] also found that the number of dives in a bout were positively correlated with the IPQ of the bout in Brünnich’s guillemots. Additionally, we found that the mean IPQ of diving bouts was higher than that of individual dives, as in studies by Elliott et al. [75] and Mori et al. [73] on Brünnich’s guillemots.

## Conclusions

We show that timing within breeding stage, and prey distributions are drivers of foraging strategies in chinstrap penguins, and found that patch quality improves with distance to the colony, but found no evidence of prey depletion near the colony increasing over time. This sheds light on how predators select foraging strategies based on the changing requirements of breeding and the spatial and temporal variability in prey distributions. While analysing the effect of population size was beyond the scope of this study, the role of population size on foraging strategies during breeding, and in particular how spatial and temporal prey availability and intraspecific competition may interact is an important avenue for future research.

## Supplementary Information

**Additional file 1.**

## Data Availability

All GPS tracking data and all TDR dive data analysed in this manuscript are available from the UK Polar Data Centre (www.bas.ac.uk/data/uk-pdc/).

## Abbreviations

GPS: Global positioning system
IPQ: Index of patch quality
LMM: Linear mixed models
TDRs: Temperature-depth recorders
