# Correction to: Foraging conditions for breeding penguins improve with distance from colony and progression of the breeding season at the South Orkney Islands

**DOI:** 10.1186/s40462-022-00330-9

**Published:** 2022-08-04

**Authors:** Jessica Ann Phillips, Annette L. Fayet, Tim Guilford, Fabrizio Manco, Victoria Warwick-Evans, Phil Trathan

**Affiliations:** 1 Department of Zoology, Oxford University, 11a Mansfield Rd, Oxford, OX1 3SZ UK; 2 Anglia Ruskin University, Cambridge Campus, East Rd, Cambridge, CB1 1PT UK; 3 British Antarctic Survey, High Cross, Madingley Rd, Cambridge, CB3 0ET UK

## Correction to: Movement Ecology (2021) 9:22 10.1186/s40462-021-00261-x

Following publication of the original article [1], it was noted that due to a typesetting error, Figs. 3 and 4 were swapped and paired with the wrong caption. The correct Figs. 3 and 4 and their captions have been included in this Correction and the original article has been corrected.

**Fig. 3:**
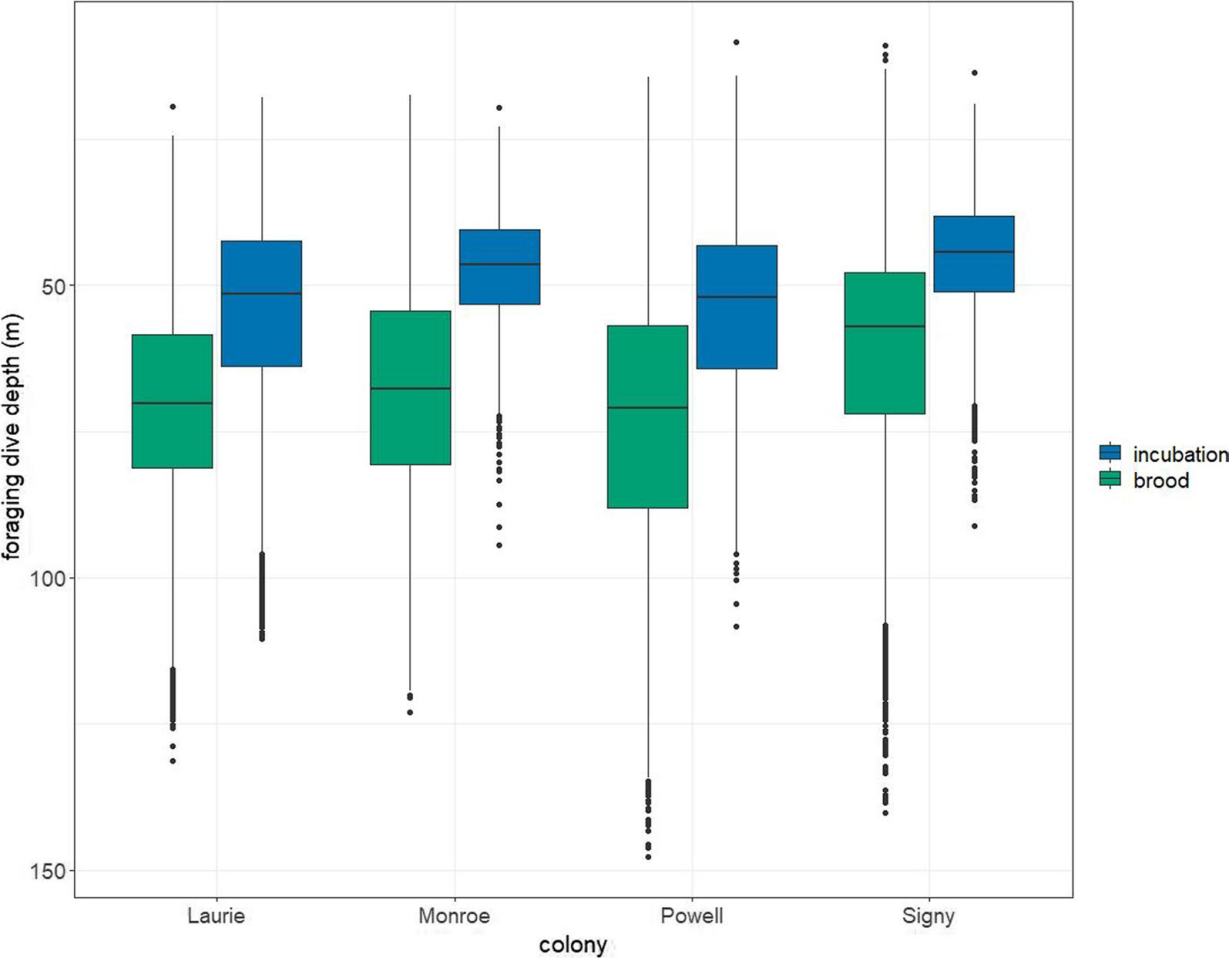
Depth of chinstrap penguin foraging dives during incubation and brood at four colonies in the South Orkney Islands

**Fig. 4:**
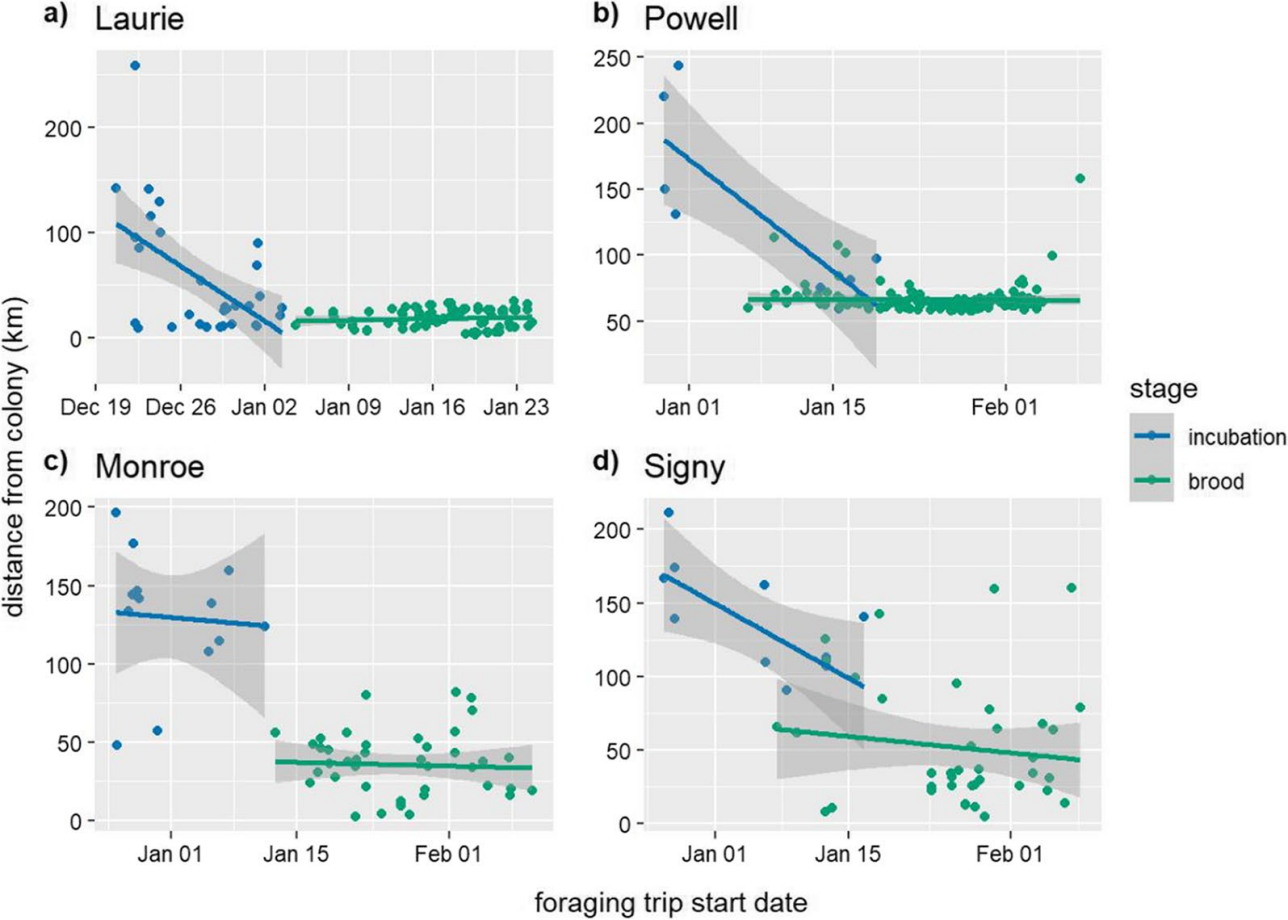
Maximum distance from the colony of foraging trips by start date during incubation and brood at **a**) Laurie Island, **b**) Powell Island, **c**) Monroe Island, and **d**) Signy Island. The shaded areas represent slope standard error

The publisher apologises to the authors and readers for the inconvenience caused by the error.
