# Supplementary material for: Foraging conditions for breeding penguins improve with distance from colony and progression of the breeding season at the South Orkney Islands
Source: Mov Ecol. 2021 May 4;9:22. doi: 10.1186/s40462-021-00261-x (PMC8094539; doi:10.1186/s40462-021-00261-x)

**Supplementary Materials**

Fig S.1 Kernel density plots of dive IPQ at a) Monroe and Laurie, and b) Powell and Signy. Dives are split into four quantiles based on distance to colony for each colony. Shapes represent 95% contour kernels, and are coloured based on the average IPQ within that quantile, where darker blues indicate higher IPQ.

a)


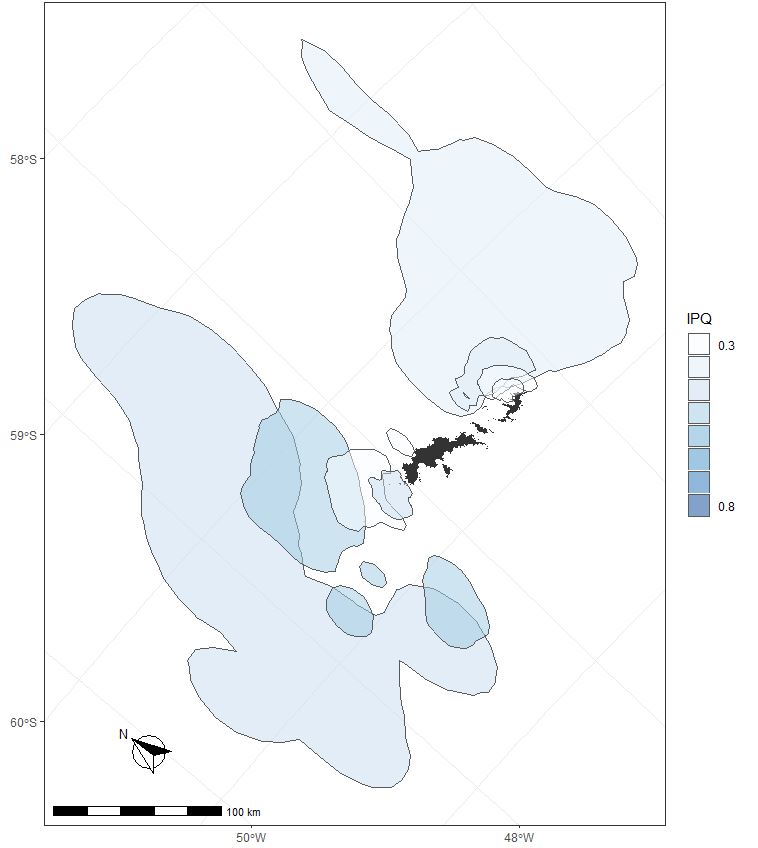


b)


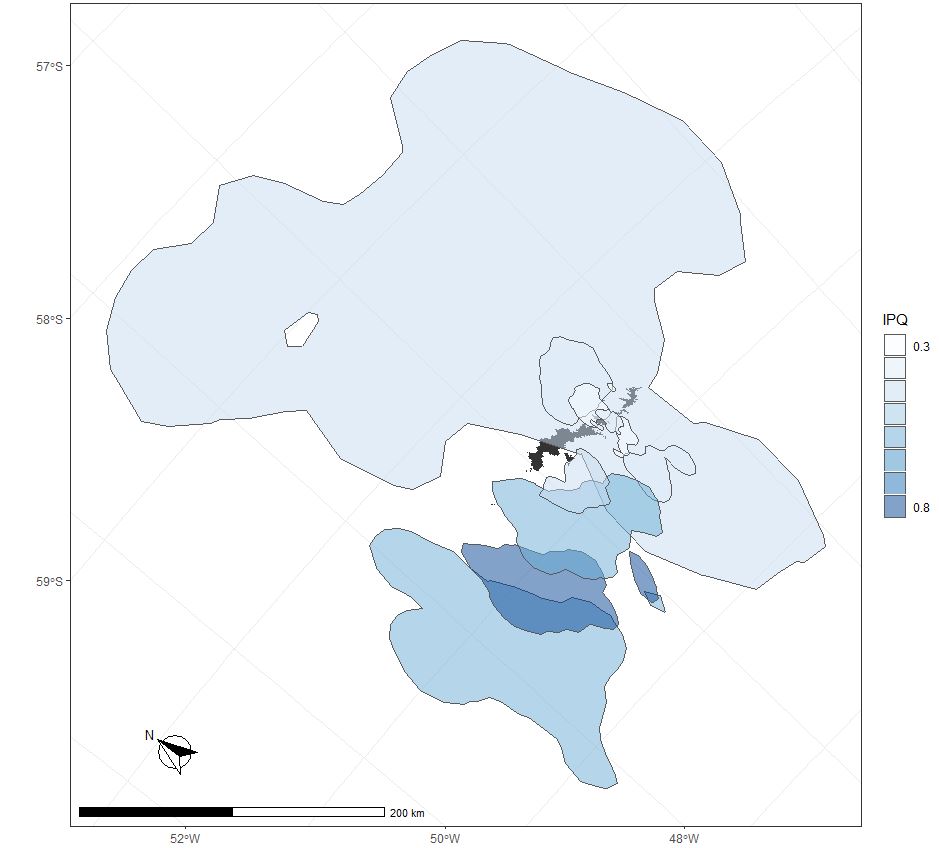

Supplement: Supplementary file 1 — Additional file 1. [file 40462_2021_261_MOESM1_ESM.docx]
